# Supplementary material for: Resolving subcellular pH with a quantitative fluorescent lifetime biosensor
Source: Nat Commun. 2022 Oct 12;13:6023. doi: 10.1038/s41467-022-33348-z (PMC9556823; doi:10.1038/s41467-022-33348-z)
Supplement: Supplementary file 7 — Reporting Summary [file 41467_2022_33348_MOESM7_ESM.pdf]

nature portfolio

Corresponding author(s): Angus P.R. Johnston

Last updated by author(s): Aug 15, 2022

Reporting Summary

Nature Portfolio wishes to improve the reproducibility of the work that we publish. This form provides structure for consistency and transparency in reporting. For further information on Nature Portfolio policies, see our Editorial Policies and the Editorial Policy Checklist.

Statistics

For all statistical analyses, confirm that the following items are present in the figure legend, table legend, main text, or Methods section.

n/a	Confirmed	
☐	☒	The exact sample size (n) for each experimental group/condition, given as a discrete number and unit of measurement
☐	☒	A statement on whether measurements were taken from distinct samples or whether the same sample was measured repeatedly
☐	☒	The statistical test(s) used AND whether they are one- or two-sided<br>*Only common tests should be described solely by name; describe more complex techniques in the Methods section.*
☒	☐	A description of all covariates tested
☒	☐	A description of any assumptions or corrections, such as tests of normality and adjustment for multiple comparisons
☐	☒	A full description of the statistical parameters including central tendency (e.g. means) or other basic estimates (e.g. regression coefficient) AND variation (e.g. standard deviation) or associated estimates of uncertainty (e.g. confidence intervals)
☐	☒	For null hypothesis testing, the test statistic (e.g. F, t, r) with confidence intervals, effect sizes, degrees of freedom and P value noted<br>*Give P values as exact values whenever suitable.*
☒	☐	For Bayesian analysis, information on the choice of priors and Markov chain Monte Carlo settings
☒	☐	For hierarchical and complex designs, identification of the appropriate level for tests and full reporting of outcomes
☒	☐	Estimates of effect sizes (e.g. Cohen's d, Pearson's r), indicating how they were calculated

Our web collection on statistics for biologists contains articles on many of the points above.

Software and code

Policy information about availability of computer code

Data collection	All fluorescent images (including lifetime information) were acquired using LAS X 3.5.7.23225. Flow cytometry data was acquired using CellCapTure v5.0
Data analysis	ImageJ scripts and image analysis was performed on ImageJ 1.53i. RStudio 1.2.5033 and GraphPad Prism 9 were used for data and statistical analyses. Flow cytometry data was analysed using FlowJo X.

For manuscripts utilizing custom algorithms or software that are central to the research but not yet described in published literature, software must be made available to editors and reviewers. We strongly encourage code deposition in a community repository (e.g. GitHub). See the Nature Portfolio guidelines for submitting code & software for further information.

Data

Policy information about availability of data

All manuscripts must include a data availability statement. This statement should provide the following information, where applicable:

- Accession codes, unique identifiers, or web links for publicly available datasets
- A description of any restrictions on data availability
- For clinical datasets or third party data, please ensure that the statement adheres to our policy

The data that support the findings of this study is available on figshare (https://doi.org/10.6084/m9.figshare.20454867.v1)

Field-specific reporting

Please select the one below that is the best fit for your research. If you are not sure, read the appropriate sections before making your selection.

- [x] Life sciences
- [ ] Behavioural & social sciences
- [ ] Ecological, evolutionary & environmental sciences

For a reference copy of the document with all sections, see nature.com/documents/nr-reporting-summary-flat.pdf

Life sciences study design

All studies must disclose on these points even when the disclosure is negative.

Sample size	No sample-size calculation was performed. A sample size of 3 was determined to be adequate as the statistical analysis showed significant differences as noted in the manuscript.
Data exclusions	No data was excluded from the analyses.
Replication	All experiments were performed at least three times independently for the figures in the main manuscript unless stated otherwise. All attempts at replication were successful.
Randomization	The well position of samples (all) and order of imaging (where appropriate for non-time series capture) were randomised.
Blinding	Blinding is not relevant to this study as there was no preconception for the response of mApple lifetime to pH, or the effect of solution addition to cells expressing mApple.

Reporting for specific materials, systems and methods

We require information from authors about some types of materials, experimental systems and methods used in many studies. Here, indicate whether each material, system or method listed is relevant to your study. If you are not sure if a list item applies to your research, read the appropriate section before selecting a response.

Materials & experimental systems

n/a	Involved in the study	
x		Antibodies
	x	Eukaryotic cell lines
x		Palaeontology and archaeology
x		Animals and other organisms
x		Human research participants
x		Clinical data
x		Dual use research of concern

Methods

n/a	Involved in the study	
x		ChIP-seq
x		Flow cytometry
x		MRI-based neuroimaging

Eukaryotic cell lines

Policy information about cell lines

Cell line source(s)	NIH-3T3, HEK293 cells were sourced from ATCC. HEK293-FT cells were sourced from ThermoFisher Scientific.
Authentication	No authentication was performed as the cells were obtained from a reliable source.
Mycoplasma contamination	All cell lines were tested monthly for mycoplasma contamination by PCR. All cell lines were negative for mycoplasma.
Commonly misidentified lines (See ICLAC register)	No commonly misidentified cell lines were used.
